# Cardiac reflections and natural vibrations: Force-frequency relation recording system in the stress echo lab

**DOI:** 10.1186/1476-7120-5-42

**Published:** 2007-11-22

**Authors:** Tonino Bombardini, Vincenzo Gemignani, Elisabetta Bianchini, Lucia Venneri, Christina Petersen, Emilio Pasanisi, Lorenza Pratali, Mascia Pianelli, Francesco Faita, Massimo Giannoni, Eugenio Picano

**Affiliations:** 1 Department of Echocardiography (Echo Lab), IFC, CNR, Pisa, Italy; 2 Digital Signal Processing Lab (DSPLAB), IFC, CNR, Pisa, Italy

## Abstract

**Background:**

The inherent ability of ventricular myocardium to increase its force of contraction in response to an increase in contraction frequency is known as the cardiac force-frequency relation (FFR). This relation can be easily obtained in the stress echo lab, where the force is computed as the systolic pressure/end-systolic volume index ratio, and measured for increasing heart rates during stress. Ideally, the noninvasive, imaging independent, objective assessment of FFR would greatly enhance its practical appeal.

**Objectives:**

1 – To evaluate the feasibility of the cardiac force measurement by a precordial cutaneous sensor. 2 – To build the curve of force variation as a function of the heart rate. 3 – To compare the standard stress echo results vs. this sensor operator-independent built FFR.

**Methods:**

The transcutaneous force sensor was positioned in the precordial region in 88 consecutive patients referred for exercise, dipyridamole, or pacing stress. The force was measured as the myocardial vibrations amplitude in the isovolumic contraction period. FFR was computed as the curve of force variation as a function of heart rate. Standard echocardiographic FFR measurements were performed.

**Results:**

A consistent FFR was obtained in all patients. Both the sensor built and the echo built FFR identifiy pts with normal or abnormal contractile reserve. The best cut-off value of the sensor built FFR was 15.5 g * 10^-3 ^(Sensitivity = 0.85, Specificity = 0.77). Sensor built FFR slope and shape mirror pressure/volume relation during stress. This approach is extendable to daily physiological exercise and could be potentially attractive in home monitoring systems.

## Introduction

**Figure 1:**
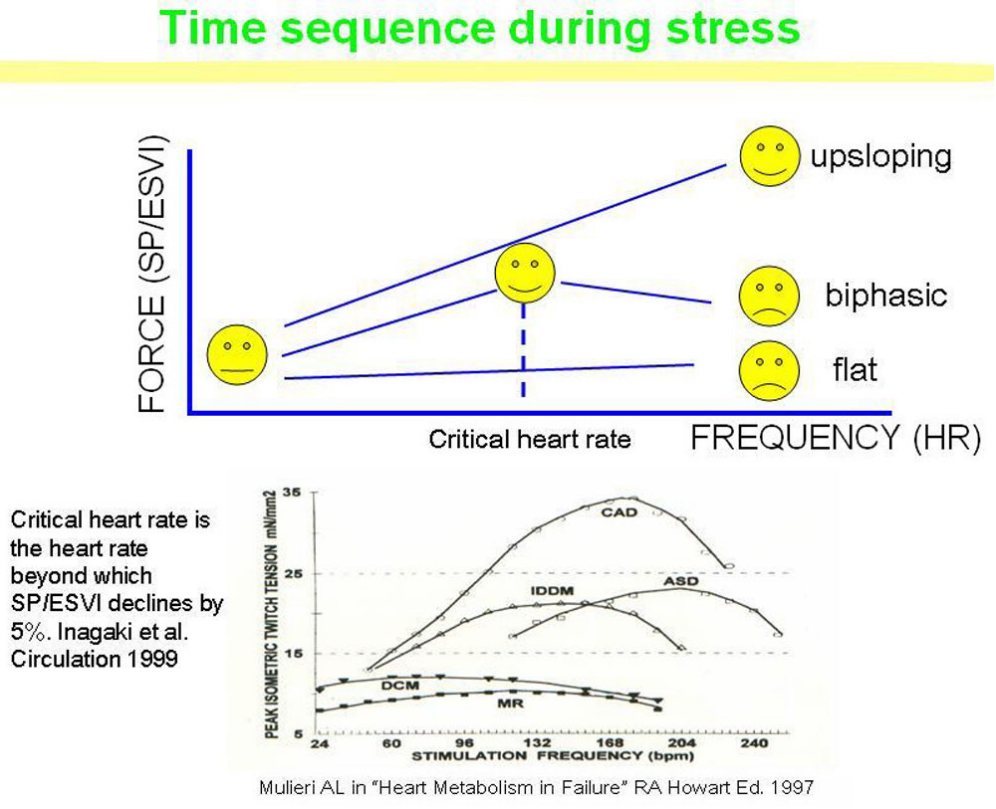
**FFR, from myocardial strips to the echo lab**. Time sequence during stress echo (upper panel). The force-frequency relation is built off line. The force-frequency relationship is defined up-sloping when the peak exercise SP/ESV index is higher than baseline and intermediate stress values; biphasic, with an initial up-sloping followed by a later down-sloping trend, when the peak exercise systolic pressure/end-systolic volume index is lower than intermediate stress values; flat or negative, when the peak exercise systolic pressure/end-systolic volume index is equal to or lower than baseline stress values. The critical heart rate (or optimum stimulation frequency) is defined as the heart rate at which systolic pressure/end-systolic volume index reaches the maximum value during progressive increase in heart rate; in biphasic pattern, the critical heart rate is the heart rate beyond which the systolic pressure/end-systolic volume index has declined by 5%; in negative pattern the critical heart rate is the starting heart rate. The critical heart rate (or optimum stimulation frequency) is the human counterpart of the treppe phenomenon in isolated myocardial strips; the optimal heart rate is not only the rate that would give maximal mechanical performance of an isolated muscle twitch, but also is determined by the need for diastolic filling. Lower panel. Plots of average steady-state isometric twitch tension versus stimulation frequency in non-failing and failing myocardium. Measurements of twitch tension in isolated left-ventricular strips from explanted cardiomyopathic hearts compared with non-failing hearts show reduction in peak rates of generation and relaxation of twitch tension and a decrease in slope of tension rate vs. contraction frequency. The FFR of these failing groups both exhibit a *negative treppe *at contraction frequencies above about 100 bpm. The contraction frequency at which the FFR begins its descending limb ("optimum stimulation frequency") declines progressively in the order: ASD (atrial septal defect), CAD (coronary artery disease), IDDM (diabetic myopathy), MR (mitral regurgitation), and DCM (dilated cardiomyopathy). (Modified from: Mulieri AL. In "Heart Metabolism in Failure" R.A. Howarth Ed. 1997. The role of myocardial force-frequency relation in left ventricular function and progression of human heart failure).

The frequency-dependent control of transmembrane Ca^2+ ^entry via voltage-gated Ca2^+ ^channels provides cardiac cells with a highly sophisticated short-term system for the regulation of intracellular Ca^2+ ^homeostasis [1]. An increased stimulation rate increases the force of contraction: the explanation is repetitive Ca^2+ ^entry with each depolarization and, hence, an accumulation of cytosolic calcium. This inherent ability of ventricular myocardium to increase its force of contraction in response to an increase in contraction frequency is known as the cardiac force-frequency relation (FFR) [2]. Its assessment is still elusive for the clinical cardiologist, although several invasive and non-invasive methods have been proposed [3-6]. Among these methods, one exploits standard 2-D stress echocardiography and another heart vibrations changes detectable by an accelerometer, i.e. a device measuring mechanical vibrations generating heart sounds [7,8]. Echocardiography uses artificially generated cardiac reflections; the accelerometer simply records naturally generated heart vibrations. With echocardiography the force is computed as the systolic pressure (SP)/end-systolic volume index (ESV) ratio [9-13]. It is important to evaluate not only the variation of force between rest and peak stress test, but also how the force varies with the increase of frequency. In fact, it is known that the FFR can be normal up-sloping or abnormal flat or biphasic, that is with an initial up-sloping followed by a later down-sloping trend [6,14] (Figure 1).

**Figure 2:**
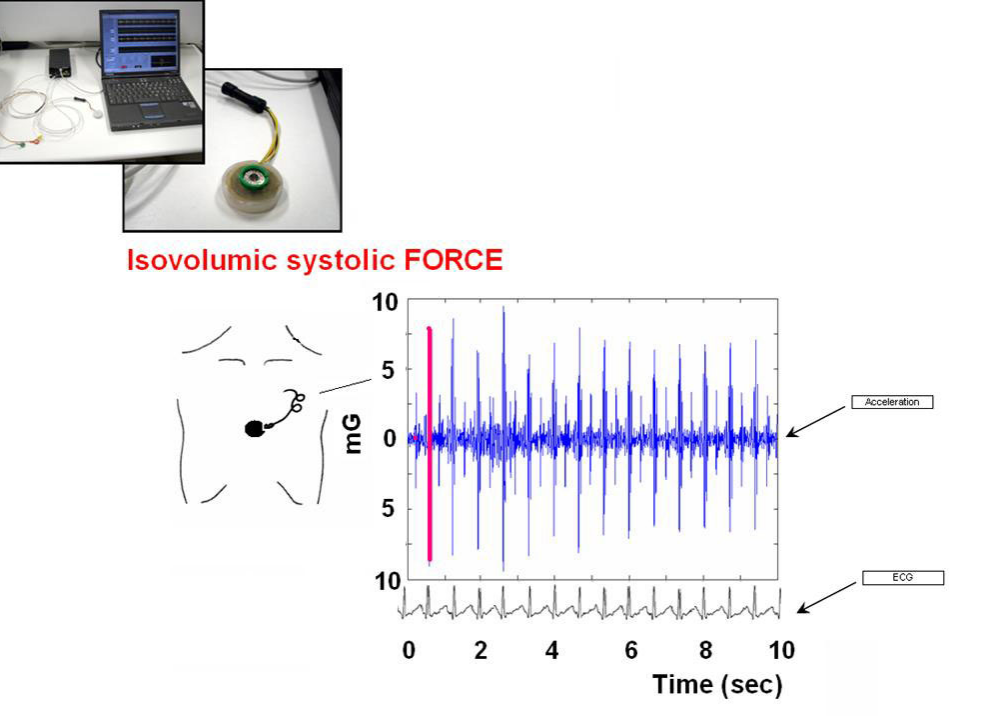
**The isovolumic contraction force sensor for FFR building.** The transcutaneous force sensor is based on a linear accelerometer. The device includes in one single package a MEMS sensor that measures a capacitance variation in response to movement or inclination and a factory trimmed interface chip that converts the capacitance variations into analog signal proportional to the motion. The device has a full scale of ±2·g (g = 9.8 m/s^2^) with a resolution of 0.0005·g. The acceleration signal is converted to digital and recorded by a laptop PC, together with an ECG signal. The system is also provided with a user interface that shows both the acceleration and the ECG signals while the acquisition is in progress. A QRS detection algorithm is used to automatically locate the beginning of the isovolumic ventricular contractions and to record isovolumic contraction force vibrations (which audible components give rise to the first heart sound). All the parameters are acquired as instantaneous values at baseline and during stress; mobile mean is utilized to assess baseline value (1 minute recording), at each incremental stress test, at peak test, and during recovery.

**Figure 3:**
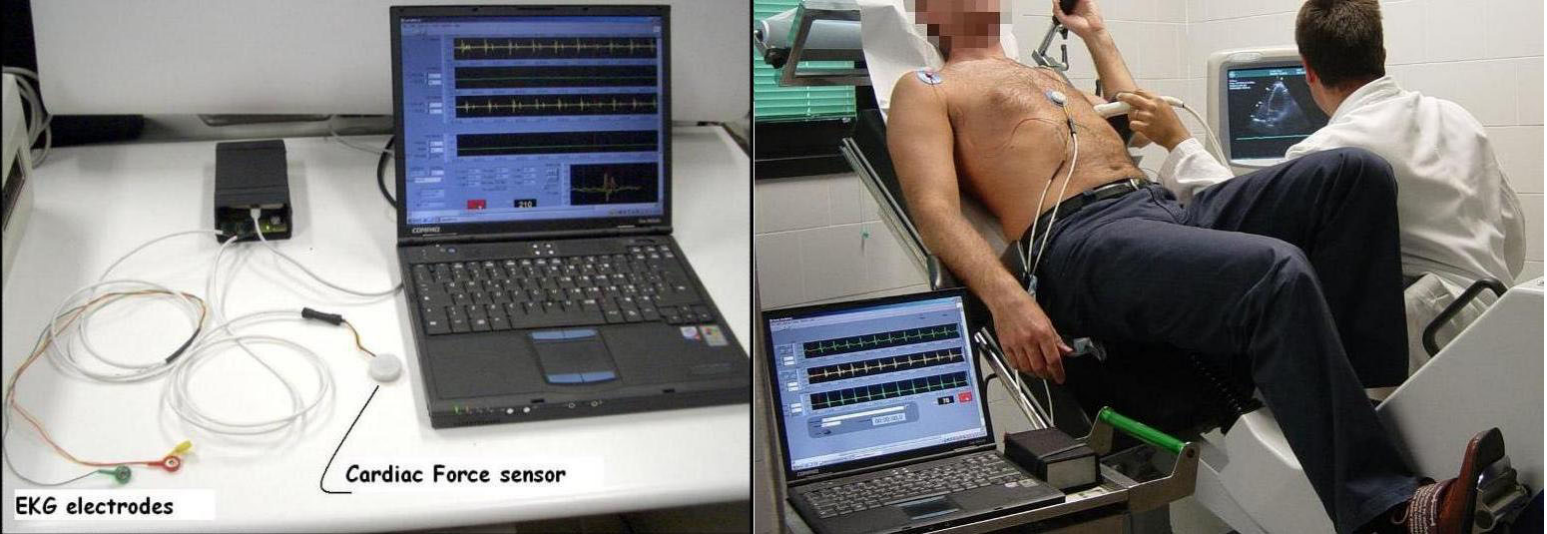
Left panel: the force-frequency relation builder device. Right panel: force-frequency recording during bycicle semi-supine stress echo. The transcutaneous force sensor was housed in a small case which is positioned in the mid-sternal precordial region and is fastened by a solid gel ECG electrode. The acceleration signal is converted to digital and recorded by a laptop PC, together with an ECG signal. The system is also provided with a user interface that shows both the acceleration and the ECG signals while the acquisition is in progress. The system is portable, and the remote transmission is feasible and simple.

**Figure 4:**
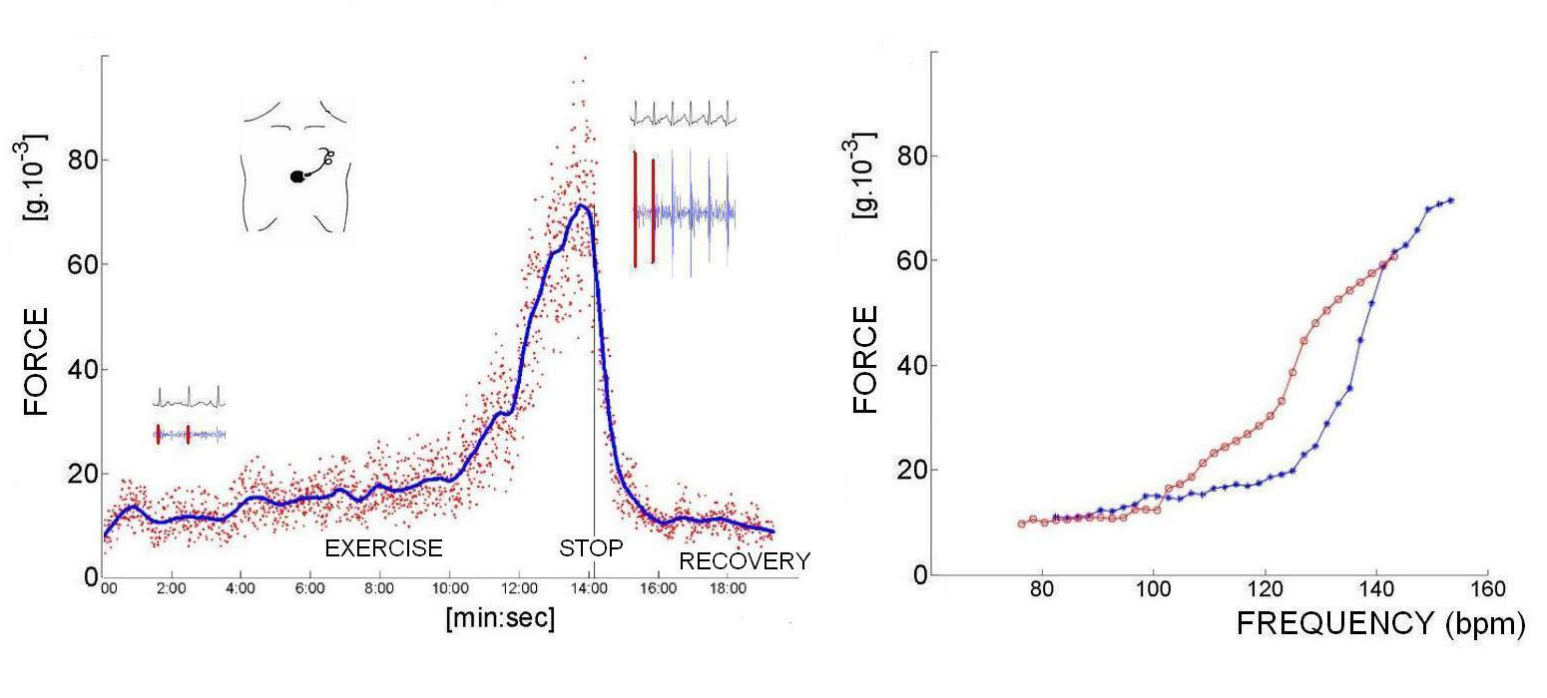
The curve of the systolic isovolumic contraction force variation as a function of heart rate is finally computed. All the parameters are acquired as instantaneous values at baseline and during stress; mobile mean is utilized to assess baseline value (1 minute recording), at each incremental stress test, at peak test, and during recovery. The data can be also read remotely by a telemetric connection. Left panel: instantaneous force values scattering (red points) depend on the respiratory cycle and thorax expansion; blue curve = force mobile mean. Right panel: blue curve = exercise in progress; red curve = recovery.

Despite the advantages of a completely noninvasive approach, the technique enables therapy effectiveness and clinical status to be verified other than by time distance checks. Since FFR slope and shape are key determinants of clinical outcome in diseased hearts, [11-13,15] FFR should be monitored with noninvasive operator-independent methods (Figure 2, 3, 4).

Aims of this study are: 1 – To evaluate the feasibility of the cardiac force measurement by a precordial cutaneous sensor 2 – To build the curve of force variation as a function of the heart rate during exercise, dipyridamole and pacing stress echocardiography. 3 – To compare the standard echocardiographic force-frequency relation measurements and hemodynamic assessment vs. this new sensor operator-independent built force-frequency relation.

## Methods

### Patient selection

We enrolled 88 consecutive patients (56 males, 62 ± 13 years) referred for stress echocardiography (49 for exercise, 31 for dipyridamole, 8 for pacing stress). The type of stress was clinically driven for exercise vs. dipyridamole, and by the presence of a permanent pace maker for pacing stress. The characteristics of the study patients are reported in Table 1. The local Ethical Committee approved the study protocol. All the patients gave their written informed consent before entering the study.

**Table 1:** Characteristics of the study patients

	EXERCISE	DIP	PACING
PT n°	49	31	8
Age (years)	58 ± 13	66 ± 11	71 ± 10
Males	33	17	6
Controls	9	0	0
Previous PTCA/By pass	15	15	2
Previous myocardial infarction	12	8	2
Arterial hypertension	27	17	5
Atypical chest pain/miscellaneous	13	10	4
BB on	21	17	5
ACEi on	11	10	5
Ca CB on	11	8	1

ACEi = angiotensin converting enzyme inhibitors; BB = beta blockers; Ca CB = calcium channel blockers

All patients met the following inclusion criteria: 1) referred to stress echo for clinically-driven testing; 2) acoustic window of acceptable quality; 3) willingness to enter the study. Exclusion criteria were: 1) unstable angina or recent myocardial infarction; 2) moderate-to-severe aortic stenosis; 3) hemodynamic instability, documentation of life-threatening ventricular arrhythmias (sustained ventricular tachycardia or ventricular fibrillation), atrial fibrillation. From the initially considered population of 92 patients, 4 were excluded for poor acoustic window (n = 2), or refusal to give written informed consent (n = 2).

### Semi-supine bicycle exercise

Graded bicycle semi-supine exercise echo was performed in 49 patients starting at an initial workload of 25 watts lasting for 2 minutes; thereafter the workload was increased stepwise by 25 watts at 2 minutes interval. A 12-lead electrocardiogram and blood pressure determination were performed at baseline and every minute thereafter [16].

### Dipyridamole stress echo

Dipyridamole stress echo was performed following the protocol of American Society of Echocardiography [16,17], using Dipyridamole 0.84 mg/kg in 6' (accelerated protocol) in 31 patients.

Contraindications of using dipyridamole were asthma, hypotension, bradyarrhythmias.

### Pacing stress echo

The study population consisted of 8 patients with a permanent pace maker. The pacing protocol was accelerated (with a 10-beat increment every 60 s) until one of the following criteria was reached: 1 – 85% of maximal heart rate (age-corrected: 220 – age for men, 200 – age for women); or 2 – PM maximal programmable heart rate (which varied widely, according to the model of PM, up to 170 bpm during stress). Stimulation was performed, wherever possible, in atrial stimulation mode, or dual-chamber (DDD) pacing to have normal contraction sequence. In the VVI-implanted patients, ventricular stimulation mode was used [10].

### Diagnostic end points and interruption criteria

The diagnostic end-points for all types of stress were: the development of obvious echocardiography positivity, obvious alterations of ECG (ST segment shift >3 mm). The exam was also stopped in case of limiting subjective side effects or hypertension (systolic pressure >220 mmHg, diastolic pressure >120 mmHg), hypotension (relative or absolute) with decrease of the blood pressure >30 mmHg, supraventricular arrhythmias (supraventricular tachycardia, atrial fibrillation), ventricular arrhythmias (ventricular tachycardia, frequent and polymorphous ventricular beats); limiting dyspnoea, or maximal predicted heart rate in the absence of ischemia [16].

### Data acquisition

All patients underwent transthoracic echocardiography at baseline and during stress.

Left ventricular end-diastolic and end-systolic volumes were measured from apical four- and two-chamber view, by an experienced observer using the biplane Simpson-method [18]. Only representative cycles with optimal endocardial visualization were measured and the average of three measurements was taken. The endocardial border was traced, excluding the papillary muscles. The frame captured at the R wave of the ECG was considered to be the end-diastolic frame, and the frame with the smallest left ventricular cavity the end systolic frame. Images were acquired at baseline and at each increase in heart rate of 10 beats during stress.

### Regional wall motion analysis

The Wall Motion Score Index (WMSI) was calculated in each patient at baseline and peak stress, according to the recommendations of the American Society of Echocardiography from 1 = normal-hyperkinetic to 4 = dyskinetic in a 17 segment model of the left ventricle [16,19].

### Blood pressure analysis

One nurse recorded blood pressures at rest and during each individual study. The blood pressure recording was made using a sphygmomanometer and the diaphragm of a standard stethoscope. Systolic and diastolic blood pressure was obtained in the right arm. During exercise test, blood pressure recording was obtained with patient lying in a left rotated semi supine position and instructed to hand grip to the left support with their left hand. Patients have been told to let their right hand go limp when blood pressure was measured.

### End-systolic pressure-volume determination

**Figure 5:**
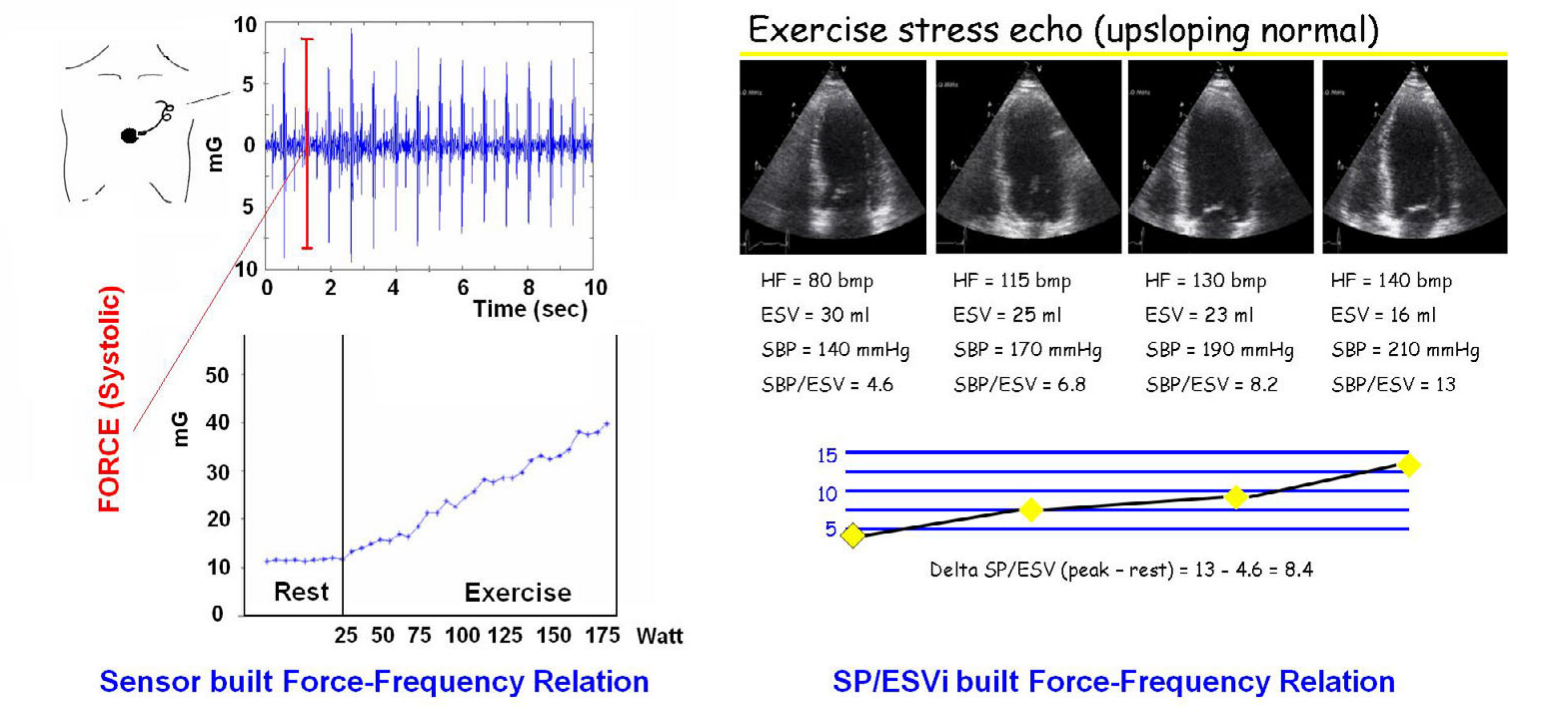
Force-Frequency relation during exercise in a normal subject wherein the memorized parameters are the heart rate, the ventricular contractile force, and the curve of force variation as a function of heart rate. Left panels: the force data is derived from the precordial cardiac sensor (maximum vibrations amplitude in the first 150 ms following the R wave). Right panels: the force data is derived from the ventricular force computed as SP/ESVi, which indicates the left ventricular end-systolic pressure volume ratio (end-systolic ventricular pressure divided by the end-systolic volume). Simultaneous recording is feasible, totally non-invasive. Echocardiography uses artificially generated cardiac reflections; the isovolumic systolic operator-independent force sensor simply records naturally generated heart vibrations.

In order to build the force-frequency relation, the force was determined at each step as the ratio of the systolic pressure (cuff sphygmomanometer)/end-systolic volume index (biplane Simpson rule/body surface area). At each heart rate step three cardiac cycles were analyzed and the ESV was calculated as the mean value. The FFR was built off line. The slope of the relationship was calculated as the SP/ESV (Systolic Pressure/End-Systolic Volume) index increase (Δ SP/ESV index from baseline to peak stress) (Figure 1 and Figure 5). The FFR was defined up-sloping when peak exercise SP/ESV index was higher than baseline and intermediate stress values; biphasic, with an initial up-sloping followed by a later down-sloping trend, when peak exercise systolic pressure/end-systolic volume index was lower than intermediate stress values [9-13]; flat or negative, when peak exercise systolic pressure/end-systolic volume index was equal to or lower than baseline stress values.

### Arterial elastance and ventricular-arterial coupling

In all, ventricular arterial coupling was indexed by the ratio of left ventricular systolic elastance index (systolic pressure/end-systolic volume index) to arterial elastance (Ea, ratio of end-systolic pressure by stroke volume). Echocardiography (for ESV and stroke volume) and cuff sphygmomanometer (systolic pressure, multiplied x 0.90 to obtain end-systolic pressure) provided the raw measurements.

Because stroke volume (and input impedance) varies directly with body size, arterial elastance was adjusted for body surface area (EaI) to better reflect differences in arterial properties with age and between the genders adjusted for differences in body size [20,21]. Of note ventricular-arterial coupling is ventricular elastance/arterial elastance, which can further be described as: end-systolic pressure/end-systolic LV volume divided by end-systolic pressure/stroke volume: the pressure terms in the numerator and the denominator cancel out, and ventricular-arterial coupling equals to stroke volume/end-systolic volume.

### Systemic Vascular Resistance (SVR)

SVR were calculated according to the traditional formula:

SVR = 80 * (MAP-5)/CO,

where 5 is an approximation of the right atrial pressure and MAP is mean arterial pressure.

### Systemic arterial compliance

Systemic arterial compliance (C) was calculated as stroke volume index/systemic arterial pulse pressure; where pulse pressure = systolic blood pressure - diastolic blood pressure [22].

### The cardiac force measurement by the precordial cutaneous sensor and the operator-independent force-frequency relation

Isovolumic myocardium contractions generate vibrations which have audible components that are responsible for the first heart sound [7,8]. These vibrations can be measured with modern accelerometer based technology. These devices are small in size and can be easily used to build a cutaneous sensor (Figure 2).

The transcutaneous force sensor is based on a linear accelerometer from STMicroelectronics (LIS3). The device includes in one single package a MEMS sensor that measures a capacitance variation in response to movement or inclination and a factory trimmed interface chip that converts the capacitance variations into analog signal proportional to the motion. The device has a full scale of ±2·g (g = 9.8 m/s^2^) with a resolution of 0.0005·g. We housed the device in a small case (Figure 3) which was positioned in the mid-sternal precordial region and was fastened by a solid gel ECG electrode. The acceleration signal was converted to digital and recorded by a laptop PC, together with an ECG signal. The system is also provided with a user interface that shows both the acceleration and the ECG signals while the acquisition is in progress. The data were analyzed by using software developed in Matlab (The MathWorks, Inc). A QRS detection algorithm was used to automatically locate the beginning of the isovolumic ventricular contractions [23]. The amplitude of the vibration due to isovolumic myocardium contraction was then obtained to record systolic force for each cardiac beat [24,25]. While echocardiography uses artificially generated cardiac reflections, the accelerometer simply records naturally generated heart vibrations, which audible components in the isovolumic (preejection) contraction period give rise to the first heart sound. Non myocardial noising vibrations (skeletal muscles, body movements, breathing) were eliminated by frequency filtering (Figure 4, 5).

The curve of force variation as a function of heart rate was finally computed as the increment with respect to the resting force value. All the parameters were acquired as instantaneous values at baseline and during stress; mobile mean was utilized to assess baseline value (1 minute recording), at each incremental stress test, at peak test, and during recovery. Baseline, peak stress, peak-rest difference as absolute value, and delta % rest-peak stress values were computed. The force-frequency relation was defined up-sloping when the peak stress force was higher than baseline and intermediate stress values; biphasic, with an initial up-sloping followed by a later down-sloping trend, when the peak stress force was lower than intermediate stress values; flat or negative, when the peak stress force was equal to or lower than baseline stress values [6,14]. The critical heart rate (or optimum stimulation frequency) was defined as the heart rate at which the force reaches the maximum value during progressive increase in heart rate; in biphasic pattern, the critical heart rate is the heart rate beyond which the force has declined by 5%; in negative pattern the critical heart rate is the starting heart rate.

### Statistical analysis

SPSS 11 for Windows was utilized for statistical analysis. The statistical analyses included descriptive statistics (frequency and percentage of categorical variables and mean and standard deviation of continuous variables). Pearson chi-square with Fisher's exact test for categorical variables and the Mann-Whitney test for continuous variables for inter-group comparisons were performed to confirm significance (using Monte Carlo method for small sample comparisons).

The one-way ANOVA was used to compare continuous variables between groups; when homogeneity of variance was not present, the Kruskal-Wallis test for nonparametric independent samples was used. Intergroup comparison was performed with Scheffe and Tamhane post hoc tests, respectively.

Relations between variables were assessed using linear regression analysis and Pearson's correlation coefficient.

Analysis of variance with Fisher's post hoc pair wise multiple comparisons was used to assess the significance of intragroup repeated measures. The best predictors of the sensor built force-frequency relation slope and shape were assessed. Receiver operator characteristic (ROC) curve analysis was used to determine potentially useful threshold values of the sensor built FFR as cut-off for normal vs. abnormal contractile reserve (as assessed by the standard echo method: Δ Peak-rest SP/ESV ≥ 4 vs. <4 in exercise, positive vs. negative FFR in Dipyridamole stress). Contingency tables were constructed to assess the sensitivity and the specificity of the new operator independent method to assess the normal threshold for contractile reserve.

## Results

### Resting and stress echocardiographic findings

Technically adequate images were obtained in all patients at baseline (by selection) and during stress.

#### At Peak Exercise

Heart rate was lower in the dipyridamole than in the exercise and pacing groups. The mean ejection fraction increased in the exercise and Dip groups, while decreased in the pacing group. Regional wall motion abnormalities occurred in 4 patients of the exercise, 1 patient of Dip and 2 patients of the pacing groups (Table 2).

**Table 2:** Rest and stress data

	**EXERCISE**	**DIP**	**PM stress**
N of pts	49	31	8
Age (yrs)	58 ± 13 §	66 ± 11	71 ± 10
Gender (M/F)	33/16	17/14	6/2
BSA (m^2^)	1.86 ± .20	1.78 ± .16	1.89 ± .25
**Standard echo measurements**			
LVMI (g/m^2^)	104 ± 30	104 ± 20	138 ± 35
HR rest (bpm)	72 ± 15	68 ± 12	71 ± 10
HR peak (bpm)	130 ± 21Δ	86 ± 15*	130 ± 12
Δ HR (rest-peak, bpm)	59 ± 20Δ	18 ± 13*	59 ± 14
LV EF % rest	59 ± 10	59 ± 10	51 ± 10
LV EF % peak	65 ± 12‡	64 ± 11*	49 ± 16
Δ LV EF % (rest-peak)	6 ± 7‡	5 ± 7	-2 ± 10
WMSI rest	1.08 ± .22	1.11 ± .24	1.21 ± .41
WMSI peak	1.10 ± .26	1.12 ± .24	1.32 ± .44
Δ WMSI (rest-peak)	.03 ± .13	.01 ± .06	.11 ± .22
**FFR with echocardiography (SP/ESVi)**			
SP/ESV index rest (mmHg/mL/m^2^)	7.6 ± 4.6	7.3 ± 2.6	6.3 ± 4.3
SP/ESV index peak (mmHg/mL/m^2^)	14.7 ± 8.4Δ	8.3 ± 3.5	10.3 ± 8.1
Δ SP/ESV index (rest-peak)	7.1 ± 5.4Δ	1 ± 2.1	3.9 ± 4
Δ SP/ESV index % (rest-peak)	103 ± 74§	13 ± 26	49 ± 37
**Sensor built FFR (isovolumic vibrations)**			
Force rest (g * 10^-3^)	10 ± 4.7	12.3 ± 6.1	9.5 ± 4.5
Force peak (g * 10^-3^)	36 ± 18.7§	15.3 ± 8.1	15.2 ± 5.6
Δ Force (rest-peak, g * 10^-3^)	26 ± 16.7§	3 ± 5	5.7 ± 4.4
Δ Force % (rest-peak)	275 ± 132§	28 ± 39	74 ± 52
**Peripheral pressures, load and coupling**			
SBP rest (mmHg)	133 ± 24	138 ± 23	141 ± 29
SBP peak (mmHg)	189 ± 26§	131 ± 27	144 ± 34
Δ SBP % (rest-peak)	45 ± 20§	-5 ± 12	2 ± 12
SVR rest (dyne * sec * cm^-5^)	2004 ± 740	2076 ± 649	2161 ± 1163
SVR peak (dyne * sec * cm^-5^)	1472 ± 464	1500 ± 636	1809 ± 737
Arterial compliance rest (mL *m^-2^/mmHg)	0.51 ± 0.19	0.48 ± 0.15	0.58 ± 0.40
Arterial compliance peak (mL *m^-2^/mmHg)	0.33 ± 0.12Δ	0.54 ± 0.20*	0.35 ± 0.19
Δ Arterial compliance % (rest-peak)	-31 ± 26Δ	18 ± 32*	-31 ± 27
Arterial elastance index rest (mmHg/mL/m^2^)	4.4 ± 1.4	4.4 ± 1.1	4.7 ± 2.7
Arterial elastance index peak (mmHg/mL/m^2^)	6.2 ± 2Δ	3.9 ± 1.3*	7.3 ± 2.9
Δ Arterial elastance index % (rest-peak)	47 ± 47Δ	-10 ± 23*	62 ± 41
Ventricular/arterial coupling rest (SP/ESV/EaI ratio)	1.7 ± .7	1.7 ± .8	1.3 ± .7
Ventricular/arterial coupling peak (SP/ESV/EaI ratio)	2.5 ± 1.3‡	2.3 ± 1.2	1.3 ± .9
Δ Ventricular/arterial coupling % (rest-peak)	48 ± 65‡	31 ± 37	-2 ± 40
Cardiac index rest (L/min/m^2^)	2.1 ± 0.6	2 ± 0.4	2.4 ± 0.8
Cardiac index peak (L/min/m^2^)	3.9 ± 1.1§	2.8 ± 0.9	2.7 ± 0.8
Δ Cardiac index % (rest-peak)	99 ± 62§	37 ± 32	14 ± 22
DBP rest (mmHg)	71 ± 11	71 ± 13	77 ± 11
DBP peak (mmHg)	93 ± 13§	68 ± 12	80 ± 16
Δ DBP % (rest-peak)	31 ± 20§	-4 ± 13	4 ± 18
LV EDV index rest (mL/m^2^)	51 ± 16	50 ± 11	65 ± 32
LV EDV index peak (mL/m^2^)	47 ± 14	50 ± 13	47 ± 30
Δ EDV index % (rest-peak)	-7 ± 16‡	-1 ± 12*	-30 ± 12

§ = significant differences between exercise and both dipyridamole and pacing stress pts; ‡ = significant differences between exercise and pacing stress pts; * = significant differences between dipyridamole and pacing stress pts. Δ = significant differences between exercise and dipyridamole stress pts.

#### Contractile response to stress

**Figure 6:**
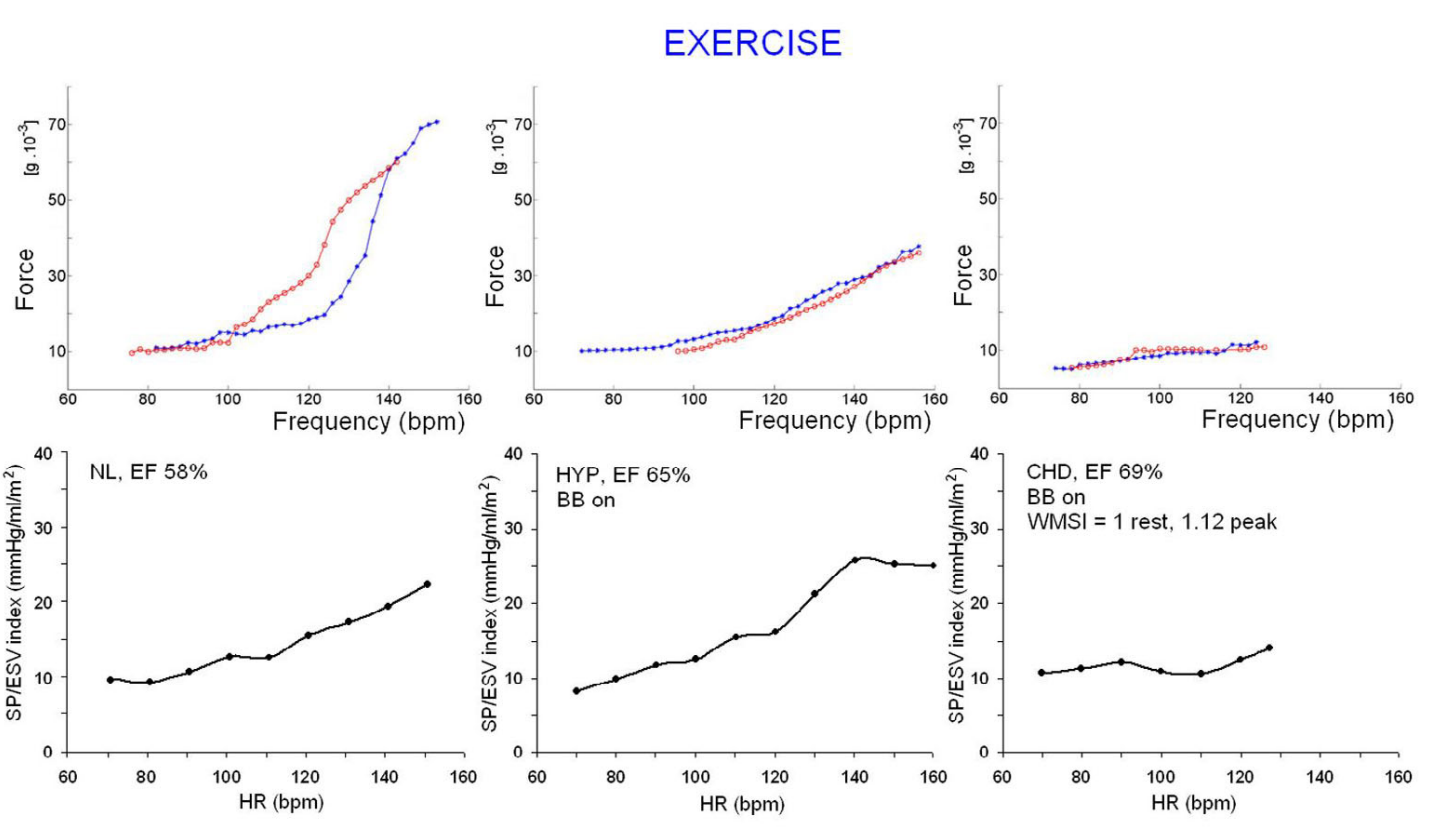
**Exercise stress echo**. Lower panels. The ventricular force is expressed as SP/ESV assessed by echocardiography in the stress echo lab. Left, force-frequency curve with stress echo in a normal subject. An increased heart rate is accompanied by an increased systolic pressure with smaller end-systolic volumes (normal up sloping force-frequency relation). The Δ rest-peak SP/ESV index is >4 mmHg/ml/m^2 ^(normal contractile reserve). Middle, force-frequency curve with stress echo in a subject with systemic hypertension with BB therapy on. The force-frequency relation is up sloping with a biphasic pattern at higher heart rates. Right, force-frequency curve with stress echo in a subject with coronary artery disease, and stress induced ischemia (abnormal force-frequency relation). The Δ rest-peak SP/ESV index is = 3, lower than 4 mmHg/ml/m^2 ^(normal cut-off for positive contractile reserve). Upper panels. Sensor built force-frequency relation simultaneously recorded in the same patients in which standard echo force-frequency relation was built. Blue curve = exercise in progress; red curve = recovery. Left, normal up sloping force-frequency relation: the Δ rest-peak force is >15.5 g * 10^-3 ^(cut-off value for normal contractile reserve). Middle, normal force-frequency relation: (Δ rest-peak force is >15.5 g * 10^-3^) but less up sloping than the control subject. Right, abnormal flat force-frequency relation: the Δ rest-peak force is 5 g * 10^-3^, much less than 15.5 g * 10^-3 ^(cut-off value for normal contractile reserve).

**Figure 7:**
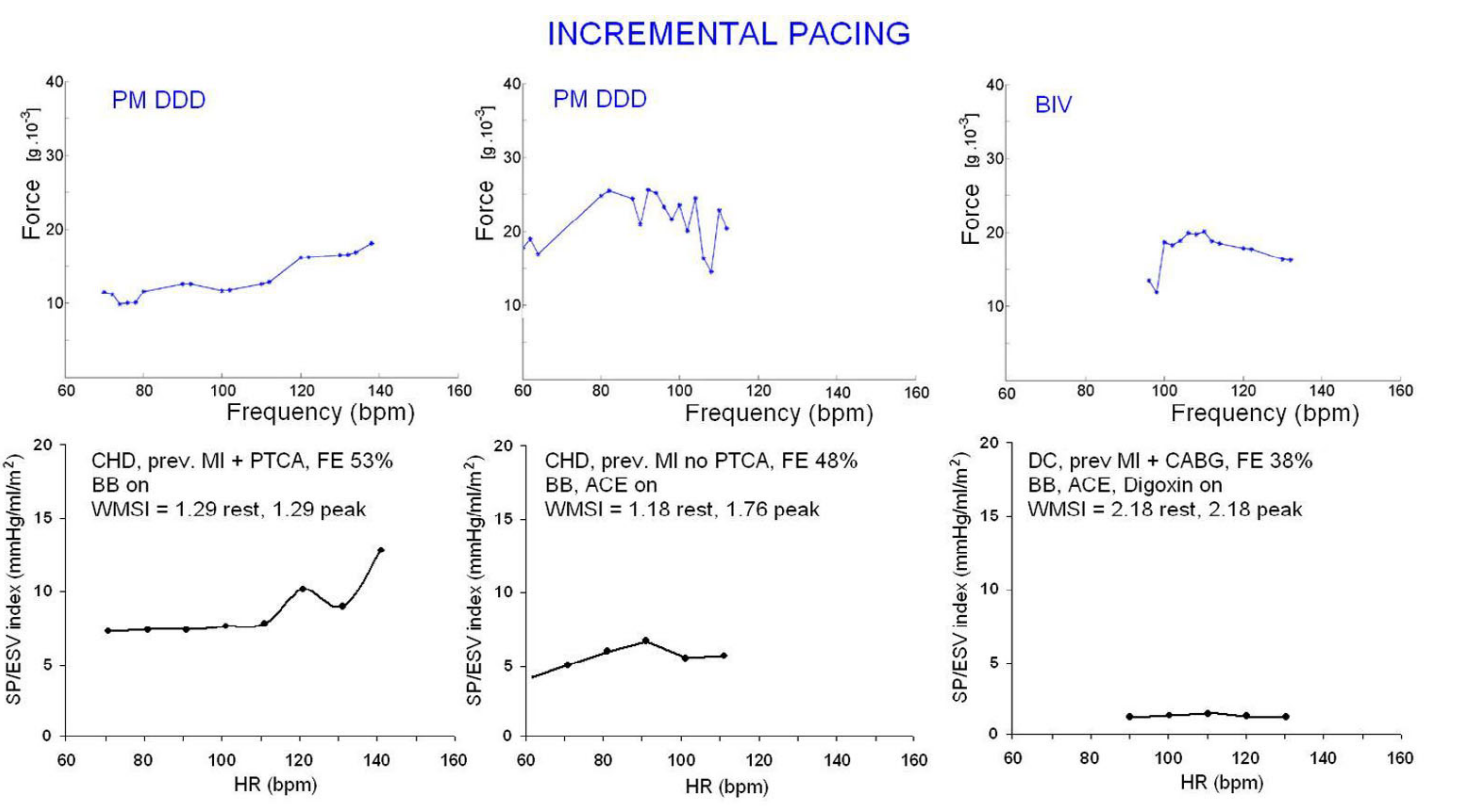
**Pacing stress.** External programming of permanent PM induces a controlled change in heart rate which is independent of the patient capability to exercise. Echocardiographic assessment is highly feasible during PM stress and technically easier than during dynamic exercise. Heart rate increase is achieved with no adrenergic stimulation which may inflate the inotropic response through a mechanism different from Bowditch treppe. Lower panels. The ventricular force is expressed as SP/ESV assessed by echocardiography in the stress echo lab. Left, residual contractile reserve; the programmed increase in heart rate is accompanied by no changes in systolic pressure with a pronounced decrease of the end-systolic volume (up sloping force-frequency relation). The Δ rest-peak SP/ESV index is >2 mmHg/ml/m^2 ^(2 mmHg/ml/m^2 ^= cut-off value for positive vs. negative contractile reserve in pacing stress). Middle, force-frequency curve with stress echo in a subject with previous MI and stress induced heterozonal ischemia. The force-frequency relation is biphasic, with an initial up-sloping trend followed by a later down-sloping trend at ischemia; an intermediate situation between normal and chronic failing hearts is effort induced ischemia, in which the normal up sloping force-frequency relation is abruptly interrupted by the hypoxic dysfunction in calcium homeostasis, resulting in reduced contractile performance at ischemia. Right, force-frequency curve with stress echo in a subject with LV dysfunction and dilation (previous MI). The force-frequency relation is biphasic, with an initial up sloping trend followed by a later down sloping trend. The Δ rest-peak SP/ESV index is = 0.1 mmHg/ml/m^2 ^(abnormal contractile reserve). Upper panels. Sensor built force-frequency relation simultaneously recorded in the same patients in which standard echo force-frequency relation was built. Left, normal up sloping force-frequency relation: the Δ rest-peak force is >7 g * 10^-3 ^(normal contractile reserve cut-off value for pacing stress). Middle, the same biphasic pattern at ischemia found with stress echo. Right, abnormal biphasic force-frequency relation; the critical heart rate occurs at 110 bpm. The critical heart rate (or optimum stimulation frequency) is the human counterpart of the treppe phenomenon in isolated myocardial strips; the optimal heart rate is not only the rate that would give maximal mechanical performance of an isolated muscle twitch, but also is determined by the need for diastolic filling.

SP/ESV index increased to 14.7 ± 8 mmHg/mL/m^2 ^in the exercise, and in the pacing (to 10.3 ± 8.1 mmHg/mL/m^2^) group, although the response was heterogeneous at the individual level (Table 2). A representation of the behavior of a normal up sloping (Δ SP/ESV index, rest-peak, ≥4 mmHg/mL/m^2^) an abnormal flat or flat-biphasic (Δ SP/ESV index, rest-peak, <4 mmHg/mL/m^2^) force-frequency relationship is shown in Figure 6 for exercise and in Figure 7 for pacing. An abnormal flat biphasic FFR was present in 13/49 pts of exercise and in 4/8 pts of pacing group.

**Figure 8:**
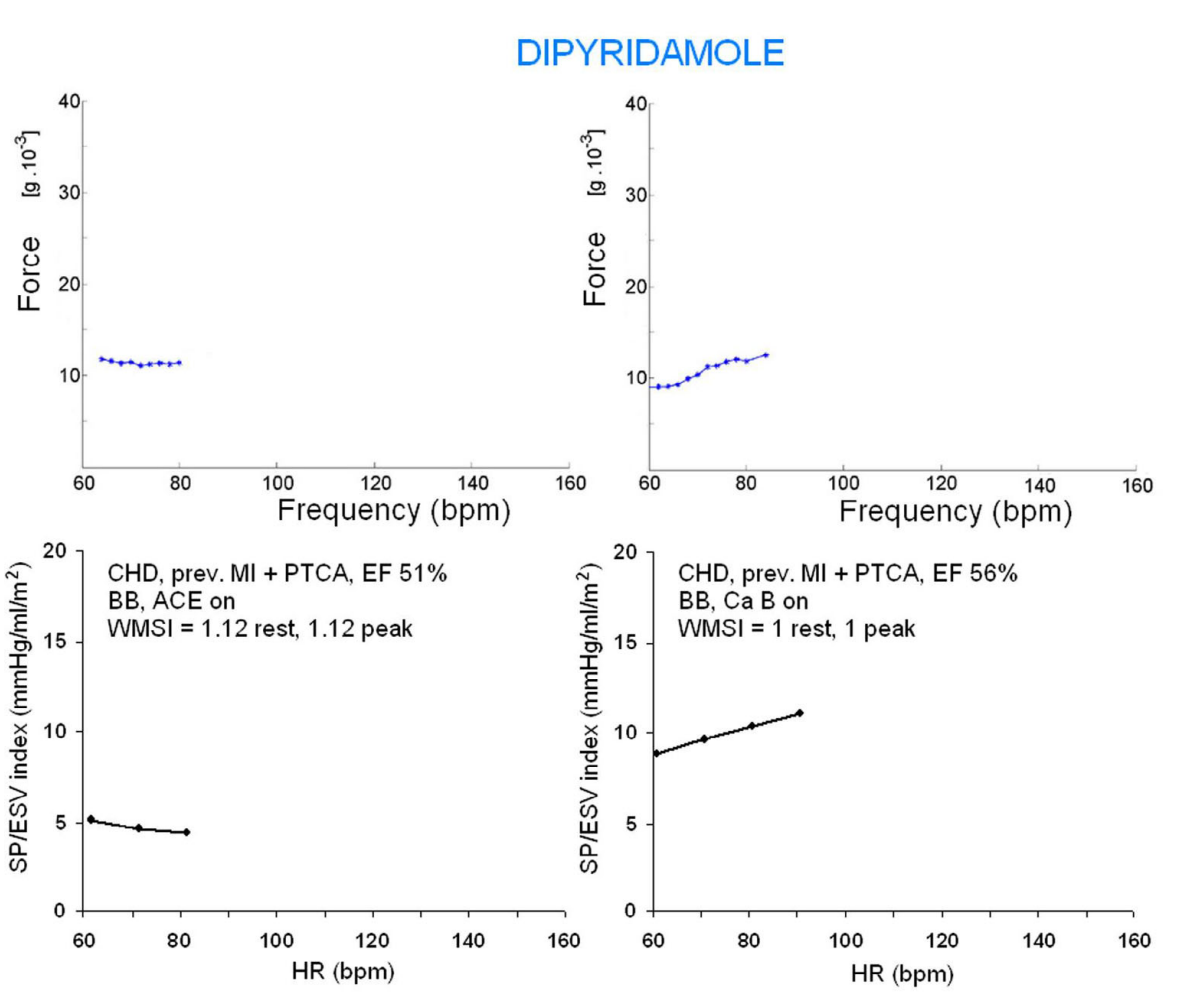
Dipyridamole stress has the well known coronary vasodilator effects mediated by the inhibition of adenosine cellular transport, eventually leading to extra cellular adenosine accumulation and steal phenomena. There is a mild catecholamine release that is responsible for the inotropic effect of the drug: normal contractile reserve is present if the force-frequency relation is up sloping instead that abnormal negative. Lower panels. The ventricular force is expressed as SP/ESV assessed by echocardiography in the stress echo lab. Left, the mild increase in heart rate at peak stress is accompanied by a mild decrease in systolic pressure without decrease of the end-systolic volume. The Δ rest-peak SP/ESV index is <0 mmHg/ml/m^2 ^(negative contractile reserve). Right, the mild increase in heart rate is accompanied by a mild decrease in systolic pressure with a more pronounced decrease of the end-systolic volume. The Δ rest-peak SP/ESV index is >0 mmHg/ml/m^2 ^(normal contractile reserve). Upper panels. Sensor built force-frequency relation simultaneously recorded in the same patients in which standard echo force-frequency relation was built. Left, abnormal negative force-frequency relation: the Δ rest-peak force is less than 0.35 g * 10^-3 ^(0.35 g * 10^-3 ^= cut-off value for positive vs. negative contractile reserve in DIP stress). Right, normal contractile reserve: the Δ rest-peak force is >0.35 g * 10^-3^.

The normal FFR cutoff was lower in the dipyridamole group (normal, Δ SP/ESV index, rest-peak, ≥0 mmHg/mL/m^2^; abnormal = Δ SP/ESV index, rest-peak, <0 mmHg/mL/m^2^) due to the lower stress heart rate increase and to the milder catecholamine increase (Figure 8). An abnormal negative FFR was present in 9/31 pts of the Dip group.

#### Peripheral pressures, load and coupling

Arterial elastance increased in the exercise and the pacing groups, while decreased in the dipyridamole, mainly due to a greater dipyridamole induced arterial compliance (Table 2).

In the whole group of patients, peak stress diastolic blood pressure was >80 mmHg in 34 patients, ≤80 mmHg in 54 patients.

Despite similar baseline values, diastolic blood pressure increased in the exercise, decreased in the dipyridamole, while unchanged in the pacing group; although the response was heterogeneous at the individual level (Table 2). An exercise induced diastolic hypertensive response (DPB ≥ 105 mmHg) was present in 10/49 patients of the exercise group; 13/31 patients of the Dip group showed a pharmacological induced hypotensive (DBP <70 mmHg) response.

#### Sensor built force-frequency relation

A consistent isovolumic systolic force signal was obtained in all patients at rest and during stress (Fig. 4, 5). In the patients as a whole, baseline force amplitude was 10.8 ± 5.3 g * 10^-3^, increasing to 26.8 ± 18 g * 10^-3 ^at peak stress; in the group of patients as a whole, the systolic force percentage increase from baseline to peak stress was significantly related to the percentage increase in heart rate (*r *= 0.63, p < 0.01).

A typical isovolumic systolic force trend during exercise, pacing and dipyridamole stress is shown in Figure 6, 7 and 8.

The force increased from 10 ± 4.7 to 36 ± 18.7 g * 10^-3 ^in the exercise, from 12.3 ± 6.1 to 15.3 ± 8.1 g * 10^-3 ^in the dipyridamole group, and from 9.5 ± 4.5 to 15.2 ± 5.6 g * 10^-3 ^in the pacing group.

Mean force percentage increase was + 275% in the exercise patients, + 28% in the dipyridamole patients and +74% in the eight pacing patients (p < 0.05 between groups) (Table 2).

In the exercise group the Δ rest-peak force was greater in the 9 control subjects than in the 40 patients (+ 45 ± 19 g * 10^-3 ^vs. + 22 ± 13 g * 10^-3^, p < 0.01).

At linear regression analysis best determinants of the force amplitude changes during stress were the age of patients, the heart rate, and the contractility SP/ESV index, both as absolute rest-peak and as rest-peak % changes (Table 3).

**Table 3:** Significant determinants of the sensor built FFR.

	Rest Force	Peak Force	FFR Δ rest-peak	FFR Δ % rest-peak
Age (yrs)		-0.53	-0.52	-0.43
BSA (m^2^)				
LVMI (g/m^2^)				
LV EF %	0.38	0.27	0.19	0.23
WMSI		-0.19		
HR (bpm)		0.58	0.63	0.57
SP/ESV index (mmHg/mL/m^2^)	0.34	0.36	0.49	0.57
Cardiac index (L/min/m^2^)		0.41	0.45	0.44
Ventricular/arterial coupling	0.38	0.21	0.18	0.22
LV EDV index (mL/m^2^)				

Multiple linear regression analysis to identify significant relationship between predictor variables (first column) and the sensor isovolumic contraction force was performed for baseline (second column) and stress values (third, fourth and fifth column). Pearson's correlation coefficients are reported in cells with significant (p < 0.05) relationships.

**Figure 9:**
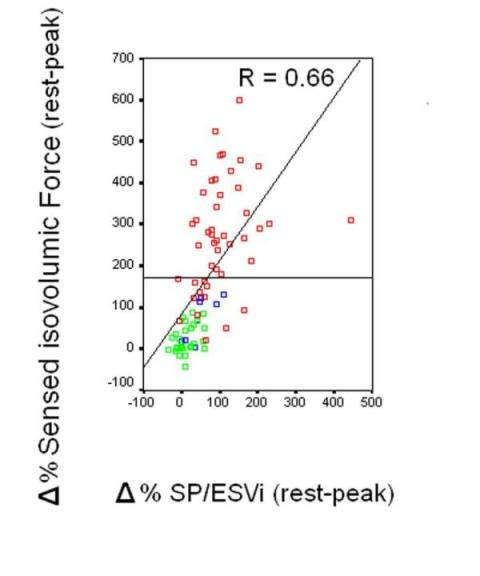
Scatter plots demonstrating relationship between sensor isovolumic contraction force (y axis) and SP/ESV index ratio (x axis) as delta % rest-peak values in the whole group of 88 patients. Green symbols: dipyridamole stress; blue symbols: pacing stress; red symbols: exercise stress. Despite cardiac vibrations propagate as mechanical shear waves, and the intervening viscoelastic thoracic tissue attenuates the higher frequencies and introduces a variable propagation delay, the sensor recorded force value shows good correlation with the standard stress-echo end-systolic elastance index (SP/ESV index ratio).

Correlations between SP/ESV index changes and force changes are displayed in Figure 9. At ROC analysis the best cut-off value of the sensor built systolic force-frequency relation for normal vs. abnormal contractile reserve at exercise or pacing stress was Δ isovolumic systolic force = 15.5 g * 10^-3 ^(Sensitivity = 0.85, Specificity = 0.77, Younden index = 1.62).

The best cut-off value for normal vs. abnormal contractile reserve at dipyridamole stress was Δ isovolumic systolic force = 0.35 g * 10^-3 ^(Sensitivity = 0.8, Specificity = 0.71, Younden index = 1.51).

## Discussion

### The sensor built force-frequency relation

A stable, reproducible, and consistent isovolumic systolic force signal was obtained in all patients. This high amplitude vibration is an expression of the tension wave front produced during initial activation of the heart in the isovolumic contraction phase [23]. Baseline force value had an ample range (from 4 to 30 g * 10^-3^). Cardiac vibrations propagate as mechanical shear waves, and the intervening viscoelastic thoracic tissue attenuates the higher frequencies and introduces a variable propagation delay [26,27]. The absolute force value in the single patient can be related to the transthoracic propagation of cardiac vibrations. In fact, when measured epicardially, preejection cardiac vibrations are up to 10 times more powerful than when measured on the chest. In our experimental model, changes of the isovolumic systolic force were linearly related to the echo SP/ESV index changes. Equally, normal vs. abnormal echo built FFR was identified by the sensor built FFR. Attempts have been made by other authors in animals and in humans to assess the usefulness of myocardial preejection vibration amplitude (which audible components give rise to the first heart sound) to quantify left ventricular contractility. Wood et al [28], studied the regional effects of myocardial ischemia on the epicardially recorded first heart sound in dogs, demonstrating that the amplitude of cardiac vibrations in the isovolumic systole are sensitive to global left ventricular contractility and independent of the site of application of the sensor (normal contracting or ischemic non-contracting area). Sakamoto et al [8] demonstrated that variations of the first heart sound amplitude are correlated to variations of the isovolumic maximum rise of LV pressure development (max. LV dP/dt).

However, to our knowledge, this is the first work in which the isovolumic systolic myocardial force vibration was utilized to build the force-frequency relation and in which, for the first time, a comparison with the echo built FFR has been made [7]. Since FFR slope and shape are key determinants of clinical outcome in diseased hearts, [11-13] FFR should be extensively monitored with this novel noninvasive operator-independent method. For sake of clarity, the conceptual and methodological differences between the FFR and the sound sensor system are contrasted in Table 4.

**Table 4:** FORCE measurement for Force-Frequency Relation building

	**Ultrasound**	**Sound**
Diagnostic vibrations	artificial	spontaneous
Imaging	yes	no
Wiggers cycle	ejection phase	isovolumic contraction
LV volumes needed	yes	no
Systolic pressure needed	yes	no
Hardware needed	2-D echo	accelerometer
Force display	off-line	on-line
Portability	difficult	simple
Remote transmission	difficult	simple

To build the FFR the memorized parameters are the heart rate, the ventricular contractile force, and the curve of force variation as a function of heart rate. The force data can be derived with ultrasound or sound methods.

### FFR and different stress

Exercise evaluates the effect of heart rate increase and inotropic reserve due to adrenergic stimulation during exercise, rather than pure heart rate effect [1,29,30].

The basic property of the force-frequency effect to progressively enhance myocardial contractility as heart rate increases is augmented at each level of increasing adrenergic stimulation as occurs in exercise by catecholamine spillover. The sensor built FFR fulfilled recording of this typical pattern, both as absolute values, and for the capability to distinguish normal vs. abnormal contractile reserve, as confirmed by the standard echo built FFR cut-off (Figure 6).

Dipyridamole has the well known coronary vasodilator effects mediated by the inhibition of adenosine cellular transport, eventually leading to extra cellular adenosine accumulation and steal phenomena [17]. There is a mild catecholamine release that is responsible for the mild inotropic effect of the drug (Figure 8). Simultaneously, the "pure" heart rate dependent increase in contractility is blunted due to the low heart rates increase during dipyridamole stress. These data are consistent with our findings; where there was a more pronounced and prolonged increase in the force signal in the exercise group vs. Dip group (Table 2). Despite the low number of patients, the pacing group showed intermediate FFR slope between exercise and dipyridamole: catecholamine levels are generally unchanged during the increase in heart rate produced by pacing [10]. Therefore, at least theoretically, pacemaker stress echo is more suitable to assess FFR rather than inotropic reserve.

### Clinical implications and current diagnostic options

The assessment of force-frequency relationship is a theoretically robust approach for evaluating left ventricular contractility, which has been deployed clinically using invasive, complex and technically demanding methods [3-6]. Previously, we proposed a non-invasive echocardiographic method [9-13] to assess the changes in contractility during exercise echo. FFR recording is feasible with a single precordial sensor fastened by a standard solid gel ECG electrode, in a totally automatic, operator independent fashion. FFR recording with a non-invasive sensor offers a new chance to monitor indexes of LV systolic function, especially in failing hearts and/or exposed to drugs [31].

**Figure 10:**
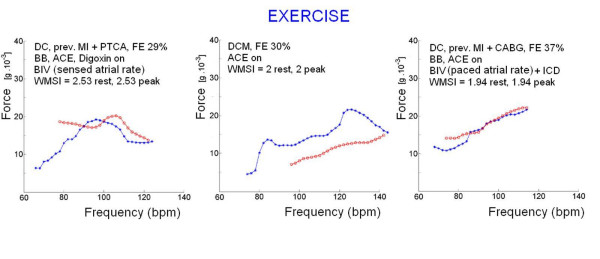
**Chronic heart failure and sensor built force-frequency relation during exercise.** Blue curve: exercise. Red curve: recovery. All patients had severely reduced LVEF% and dilated left ventricle. Left and middle panel: the force-frequency relation is biphasic, with an initial up sloping trend followed by a down sloping at critical heart rate (left panel, critical heart rate = 90 bpm; middle panel, critical heart rate = 130 bpm). Right panel: the upper rate of the paced heart rate is 110 bpm, and the negative down sloping of the biphasic FFR is avoided.

**Figure 11:**
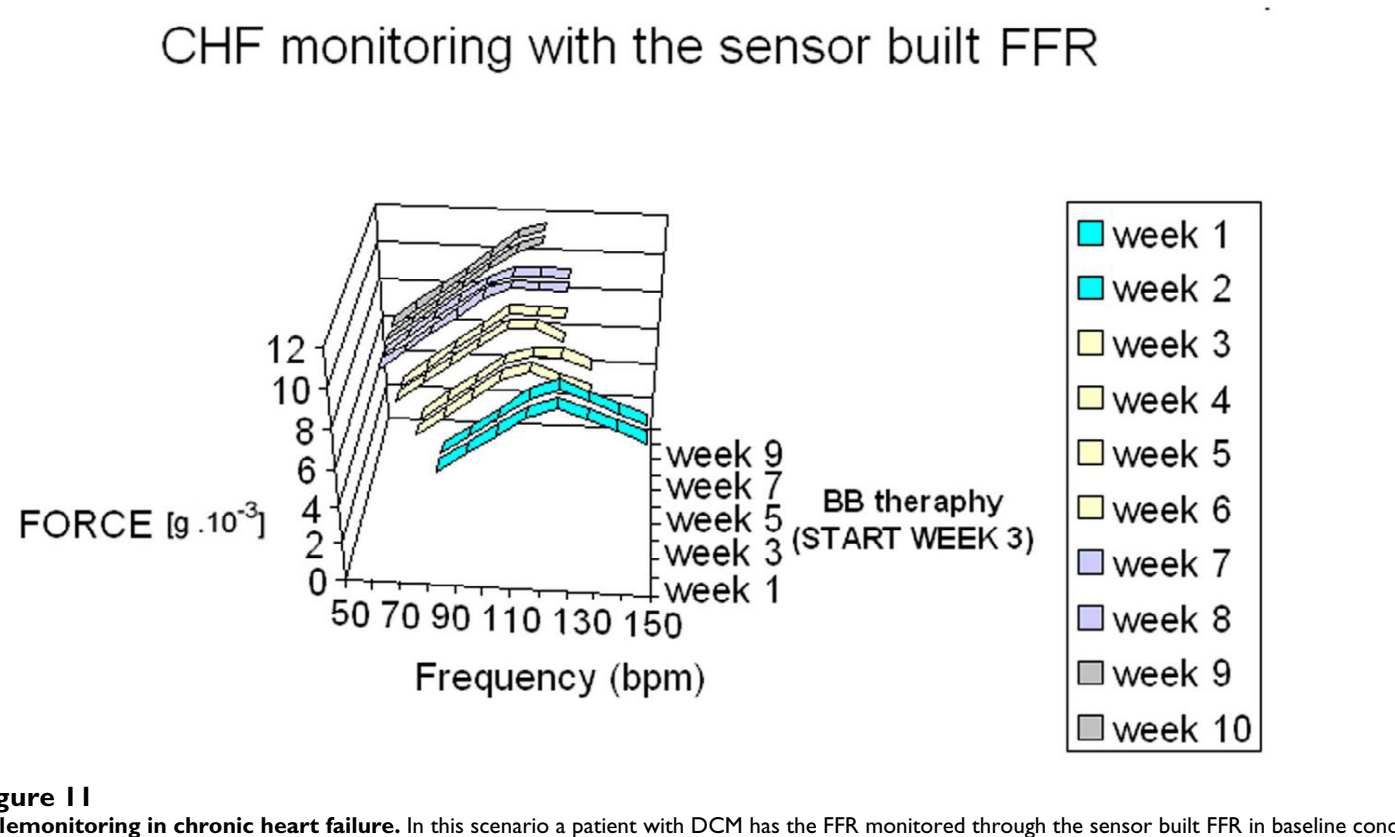
**Telemonitoring in chronic heart failure.** In this scenario a patient with DCM has the FFR monitored through the sensor built FFR in baseline conditions (week 1) and during beta-blockade titration (weeks 2 to 9). The force-frequency relation of these diseased hearts exhibits a flat or negative slope at contraction frequencies above about 100 bpm. Therapy with beta blockers has proved effective in cardiomegaly regression, however patients do not respond uniformly. The bradycardic action of beta blockers reduces the number of daily working phases of the heart in the negative part of the force-frequency relationship. To optimize chronic heart failure therapy, monitoring the patient's FFR enables the effectiveness of therapies to be verified. By monitoring in accordance with this new sensor, not only can positive or negative decompensation changes as a result of certain events be determined, but also the variation in the force-frequency curve over twenty-four hours, days or weeks. A three-dimensional diagram could be determined which for each heart rate not only indicates the instantaneous force value but also enables the variation of said value with time to be monitored.

**Figure 12:**
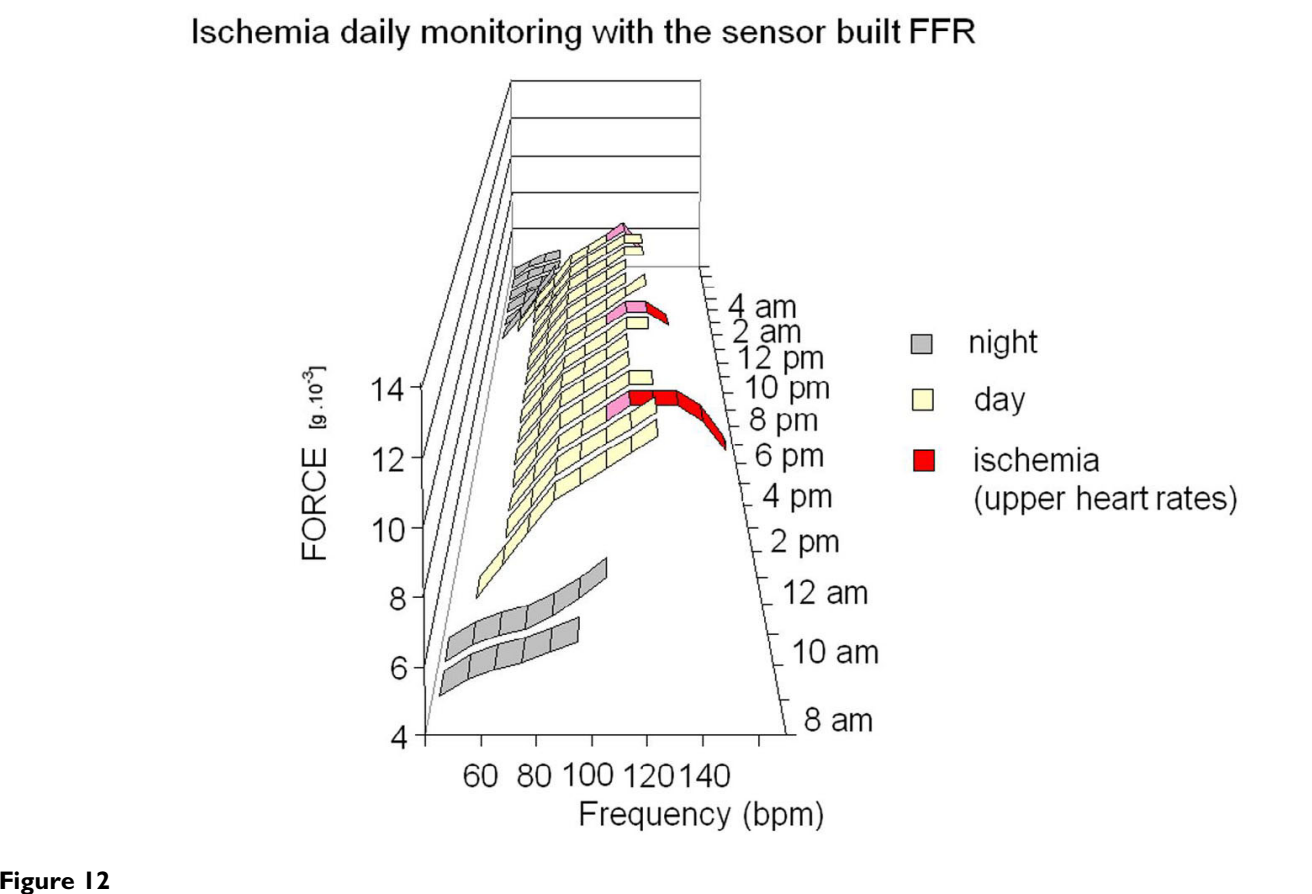
**Telemonitoring in coronary artery disease**. An intermediate situation between normal and chronic failing hearts is effort induced ischemia, in which the normal up sloping force-frequency relation is abruptly interrupted by the hypoxic dysfunction in calcium homeostasis, resulting in reduced contractile performance at ischemia. In this scenario a three-dimensional diagram could be determined in which for each heart rate not only indicates the instantaneous force value but also enables the variation of said value with time to be monitored. With patient sleeping (grey FFR curves) range of frequencies of the FFR are reduced and the slope of the relation flattened do to the reduced catecholamine spillover. During daily activities, the range of frequencies of the FFR are expanded and the slope of the relation steeper. At higher heart rates induced ischemia (red symbols), immediate blunting of the FFR occurs and a sudden descending limb begins.

In diseased hearts the force-frequency relation is altered. As the heart fails, there is a change in the gene expression from the normal adult pattern to that of fetal life with an inversion of the normal positive slope of the force-frequency relation: systolic calcium release and diastolic calcium reuptake process is lowered at the basal state and, instead of accelerating for increasing heart rates, slows down [12]. The force-frequency relation of these diseased hearts exhibits a flat or negative slope at contraction frequencies above about 100 bpm [14,15] (Figure 10). The heart rate at which the contractile force begins to decline diminishes progressively for known pathologies such as ischemic cardiomyopathy, diabetic cardiomyopathy, mitral regurgitation and dilated cardiomyopathy. The optimum contraction rate, i.e. the rate corresponding to the strongest contractile force, varies for each pathology, for each different stage of the illness, and for each patient [23]. Different therapies are known to treat chronic heart failure, however the response must be tailored to each patient. For example, therapy with beta blockers has proved effective in cardiomegaly regression and in improving the myocardial function in patients suffering from dilated cardiomyopathy, however patients do not respond uniformly to this therapy. The bradycardic action of beta blockers reduces the number of daily working phases of the heart in the negative part of the force-frequency relationship. As the flattening and the descending limb of the force-frequency curve appears at different frequencies in the different types of cardiac decompensation and in different patients, the effectiveness of the action of beta blockers is variable. To optimize chronic heart failure therapy, it therefore appears essential to identify in each patient the ascending part of the force-frequency curve and the specific rate which, when exceeded, initiates the flattening and the descending part, in order to optimize individual therapy for chronic heart failure [6]. Monitoring the patient's conditions with this new sensor built FFR not only enables his condition to be verified in terms of identifying an abnormal situation not in itself critical but predictive of a future worsening of chronic heart failure, but also it enables the effectiveness of therapies and their influence on the patient's condition under conditions of normality to be verified [31] (Figure 11). As distribution of the daily activities of the patient is variable, during the day the patient experiences, depending on the activities normally performed, periods in which his force-frequency relationship is of positive slope and periods in which his force-frequency relationship is of negative slope or flat. Known check up systems do not enable therapy effectiveness to be verified other than by occasional checks on the patient under rest conditions. By monitoring in accordance with this new sensor, not only can positive or negative decompensation changes as a result of certain events be determined, but also the variation in the force-frequency curve over twenty-four hours, days or weeks can be evaluated. A three-dimensional diagram could be determined which for each heart rate not only indicates the instantaneous force value but also enables the variation of said value with time to be monitored. (Figure 12). If the curve of force variation at a determined heart rate during the course of the day is reproduced as a two-dimensional diagram, it gives a continuous indication of the progress of decompensation situations in response to the therapies adopted.

## Limitations of the study

### Heterogeneity of the study population

The patient's number and the incoming disease were different in the exercise, dipyridamole and pacing stress groups. Controls were present only in the exercise group: it isn't possible to have physiological and hemodynamic comparisons between stress groups. However, aim of this study, was not to assess physiological patterns and differences between type of stress, that are well known, but simply assess the feasibility of sensor isovolumic contraction force recording during stress echocardiography, and to compare the standard operator-dependent echo vs. this sensor operator-independent built force-frequency relation during stress.

### Pressure measurements

Because only non-invasive measurements were available, systolic cuff pressure was used as a surrogate for end-systolic pressure. The non-invasive results tend to overestimate the actual ratio, in large part because the brachial arterial pressure reflects an amplified LV pressure wave secondary to the rheological properties of the arterial tree, and this difference may lead to overestimation of left ventricular pressure, specially if younger subjects are studied, in which blood pressure tends to be overestimated [32]. However, the peak systolic pressure/end systolic volume ratio is a reproducible method of assessing left ventricular performance during exercise [33]. Several experimental studies in animals [34] and in human beings [35] have shown that use of peak systolic pressure yielded nearly identical pressure-volume relations.

### Contractility

The estimation of the LV systolic elastance index by SP/ESV index assumes V_0 _= 0. The volume axis intercept of the end-systolic pressure/volume relationship V_0 _is generally lower in normal subjects than in CHF patients, and may also differ among DCM patients. Nevertheless, SP/ESV index is generally considered as a valid approximation of LV end systolic elastance [36]. This may not hold true during exercise if V_0 _changes. It has been reported that V_0 _is not appreciably altered by inotropic stimulation [37] or changes in loading conditions: Maughan et al [38] showed that V_0 _changed by only 5 mL on average in response to changes in peripheral vascular resistances of 50 to 200% of normal; Little and Cheng [39] observed no significant change in V_0 _during treadmill exercise in dogs. It is likely, however, that V_0 _changes during exercise but this is impossible to assess at such levels of exercise in man.

### Lack of comparisons with invasive measurements of force-frequency relation

Since end-systolic elastance (Ees), expressing the slope of the in-vivo, end-systolic ventricular pressure vs chamber volume relation, is the most "foolproof" window into in vivo myocardial contractility, Ees should be measured at each heart rate step increase, as made by Liu and coworkers [3]. But measuring Ees for increasing heart rates is impractical: increasing heart rates obtained with temporarily pacing has to be adjunct to the LV conductance catheter, the LV pressure catheter, the vena cava balloon, and to afterload changes. However, at least cath lab animal studies should be done to validate the noninvasive, operator independent, sensor builded FFR.

### Positive vs. negative contractile reserve and medications

Medication differences may have had an impact, blunting the force-frequency relation and the diastolic blood pressure. However, this variable could not be controlled for ethical and practical reasons. In addition, the index is relatively independent of afterload changes, since for a given level of contractility – blood pressure reduction should be accompanied by smaller end-systolic volume values.

## Conclusion

Force-frequency recording is feasible with single precordial vibrations sensor connected to a standard ECG monitoring electrode and quantitatively documents left ventricular contractility changes in a totally automatic, operator independent fashion.

Sensor built force-frequency relation is strictly related to the standard stress echo built FFR. Echocardiography uses artificially generated cardiac reflections. The isovolumic systolic force sensor simply records naturally generated heart vibrations. The FFR is built on line. The system is portable, and the remote transmission of the FFR slope and shape is feasible and simple.

To establish fully the advantages and limits of this new method, comparisons with left ventricular pressure-volume loops in humans and multicenter study data are needed.

## Abbreviations

C = systemic arterial compliance

CO = cardiac output

DCM = dilated cardiomyopathy

DC = dilated ischemic cardiomyopathy

EaI = effective arterial elastance index

EF = ejection fraction

EDV = end-diastolic volume

ESV = end-systolic volume

FFR = force-frequency relation

g = acceleration unit (9.8 m/sec^2^)

HR = heart rate

LV = left ventricle/ventricular

SP = systolic pressure

SVR = systemic vascular resistance

## Authors' contributions

T.B. conceived this study, performed the data analysis, and drafted the manuscript; L.V., C.P., E.Pa., L.P. and M.P. were responsible for data collection and revised the manuscript; V.G., E.B., F.F. and M.G. were responsible for technology development and digital signal processing ; E.Pi. gave a contribution to preparation of study design, data discussion, and critical revision of the manuscript.
